# Grammar tests, de facto policy and pedagogical coercion in England’s primary schools

**DOI:** 10.1007/s10993-020-09571-z

**Published:** 2020-12-12

**Authors:** Ian Cushing

**Affiliations:** Department of Education, Brunel University London, London, UK

**Keywords:** Language testing, Language ideology, Schools, England, Education policy, Policy enactment

## Abstract

Since their introduction by the Conservative government in 2013, primary school children in England have taken a mandated grammar, punctuation and spelling assessment, which places an emphasis on decontextualised, standardised English and the identification of traditional grammatical terminology. Despite some concise criticisms from educational linguists, there remains no detailed and critical investigation into the nature of the tests, their effects on test takers, and the policy initiatives which led up to their implementation. This article contributes to this gap in knowledge, using critical language testing as a methodological framework, and drawing on a bricolage of data sources such as political speeches, policy documents, test questions and interviews with teachers. I discuss how the tests work as de facto language policy, implemented as one arm of the government’s ‘core-knowledge’ educational agenda, underpinned by a reductive conceptualisation of language and a problematic discourse of ‘right/wrong’ ways of speaking. I reveal how teachers talk about the ‘power’ of the tests, intimidating and coercing them into pedagogies they do not necessarily believe in or value, which ultimately position them as vehicles for the government’s conservative and prescriptive language ideologies.

## Introduction

Since 2013, 10–11 year-old students in England take an annual mandated assessment in Grammar, Punctuation and Spelling (GPS) in their sixth and final year of primary school. Sat by around 600,000 students each year (Department for Education [DfE] 2019a) as part of an assemblage of Standardised Assessment Tests (SATs) along with maths, phonics and reading comprehension, the GPS assessments are a key component of the Conservative government’s post-2010 education reforms. These reforms place a nostalgic (re)emphasis on ‘traditional’ grammar and language study, with a particular focus on the identifying and labelling of grammatical constructions, the explicit requirement for teachers to model standardised English and the privileging of formal written language over talk (see Cushing 2020a). SATs and the GPS tests have attracted criticism from teachers, academics, trade unions and anti-test campaign groups, arguing that they place undue pressure on young people and bear little relevance to ‘real life’ language, culminating in school strikes, test boycotts and grassroots-led government inquires (see Mansell 2017). Adopting a critically orientated and discursive approach, this article explores the ways the GPS tests work as a de facto language policy mechanism (e.g. Shohamy 2001) at the epicentre of curriculum reform. I trace the political and language ideological discourses which framed their implementation by government, and explore the impacts and consequences of the tests on teachers’ lives. The article is guided by two research questions:

1. What language ideologies are embedded in the GPS tests, their associated educational policies and the political metalinguistic discourses surrounding these?
2. How do the GPS tests work as de facto language policy, in their ability to regulate and manipulate language pedagogies and ideologies in schools?

These questions are explored using analytical tools for critical language testing as outlined in Shohamy (2001), tracing metalinguistic discourses about the tests across policy layers (e.g. Barakos and Unger 2016) and highlighting connections between teachers’ lived experiences, classrooms and broader socio-political contexts. I draw on a bricolage of data to do so: the GPS test papers and assessment criteria, guidance for test creators, curriculum documents, political discourse, government reports, surveys and interviews with teachers. The range of data allows me to capture both ‘policy as text’ and ‘policy as discourse’ (Ball 1993: 10), exploring the technical aspects of the tests, the national educational policy context in which they exist, and their social and professional consequences. I take language tests to be part of language policies, as one mechanism of how language ideologies come to materialise and as one attempt to regulate language use (e.g. Frost and McNamara 2018; Menken 2008; Shohamy 2001, 2007, 2008). I argue that the tests embody an overt propagation of language ideologies, defined as ‘sets of beliefs’ about language which ‘envision and enact ties of language to identity, to aesthetics, to morality, and to epistemology’ (Woolard 1998: 3). In particular, I show how the tests and their enactment can reproduce the standard language ideology (Milroy 2001), whereby the standardised form of a language is constructed as ‘better’ or ‘more valuable’ than nonstandardised variants. I show how the tests are deployed by government as an attempt to impose constraints on what kind of language work takes place in classrooms. I show how the GPS tests are used by policy makers to construct claims about raising literacy standards, and how teachers report feelings of being coerced and intimidated into language pedagogies that they do not value. The agency of teachers within the language policy cycle is thus a key reference point within the discussion that follows (Menken and García 2010). The following section outlines some contextual details in relation to tests and language ideologies in England’s schools, and how the GPS tests fall into this historical continuity.

## Testing, pressure and standardised language benchmarks in England’s schools

Formal testing is a prominent part of school life in England, with a marked and gradual increase in statutory tests anchored to the introduction of a state-mandated, national curriculum in 1988 (Wyse and Torrance 2009). First introduced into English schools in the 1990s, Standardised Assessment Tests (SATs) have increased teacher accountability through the public availability of attainment data and become a key technology of the ‘standards discourse’ which permeates contemporary neoliberal educational policy in both England and the USA (e.g. Moss 2017; Menken 2008). Pratt’s (2016) history details how, through both Conservative and Labour governments, SATs have come to play a central role in England’s schools, tracing the slow but relentless process of increased teacher surveillance and accountability (see also Page 2017; Marshall 2017). Critics of SATs have argued that they place extreme pressure on young people (e.g. Bradbury 2019), work as a policy compliance tool which deprofessionalises teachers in coercing them into overtly test-focused pedagogies (e.g. Braun and Maguire 2018) and symbolise a market-driven education policy concerned with data, international standings and competition over student and teacher welfare (e.g. Ball et al 2012; Ozga 2009). This focus on testing and performance is generally at odds with primary school cultures in England which have traditionally emphasised play, pastoral care and creative expression (Roberts-Holmes and Bradbury 2016). Working at a counterpoint to these policy and pedagogical tensions are material threats to schools, with the possibility of government intervention and new management impositions if they do not meet benchmark standards, with SATs data being one of the indicators Ofsted^1^ use to rate the ‘quality’ of schools during inspection visits.

The current iteration of SATs, which includes the GPS tests, are one part of post-2010 curriculum and assessment reforms in England, characterised by a neoconservative policy nostalgia for ‘traditional’ education such as the explicit appreciation of ‘correct’, standardised English and canonical, British literature (see Cushing 2020b; Yandell 2017). It is important to foreground that standardised English has long been used by successive Labour and Conservative governments as a proxy for societal standards (Cameron 2012; Crowley 2003). Here then, the GPS tests are framed as a continuity of a longer historical narrative in which governments have attempted to control and regulate language policies and pedagogies in schools (e.g. Whetton 2009; Wyse and Torrance 2009). In addition to the GPS tests, the current SATs assemblage in England requires primary school students to take externally marked assessments in phonics, reading comprehension and mathematics, and an internal assessment in writing which places a strong emphasis on grammar. Existing critiques of the GPS tests (e.g. Barrs 2019; Rosen 2015; Safford 2016) have focused on how they are built on a reductive version of language knowledge which is divorced from social reality, emphasising the decontextualised, labelling of grammatical terms and uncritically promoting the standard language ideology. In this article I develop this work by employing tools and concepts from critical language policy and testing, exploring the negative washback effects that tests can have (e.g. Au 2011; Stobart 2008; McNeil 2000) and their influence on teacher agency and autonomy. In the following section, I further align the work with the aims and methods of critical language testing, before describing the methods used to generate the data.

## Critical language testing

This research is aligned with the aims and methods of critical language testing, which sees tests as non-neutral products of political, ideological, educational and social agendas, deployed as disciplinary tools by authoritative bodies to control curricula and knowledge (e.g. Blackledge 2009; Shohamy 2001, 2006, 2007, 2008; Spolsky 1995). Tests are not isolated and detached acts but are a product of language ideology and one mechanism of a language policy, anchored in the social life of communities (McNamara and Roever 2006). The critical exploration of language tests then, requires a discursive approach to exploring policy enactment (e.g. Barakos and Unger 2016; Wodak and Savski 2018), focusing on the felt impact, affect and consequences of tests on those that take them in addition to the design of the tests themselves. As is the case with other policy mechanisms, tests are not simply ‘implemented’, but are ‘enacted’ by policy agents in various ways, which may involve negotiation, resistance and varying degrees of policy compliance (Ball et al 2012).

Tests have life-changing implications for teachers and students as well as their power to manipulate educational systems. Shohamy (2001, 2006) identifies three ways in which tests might do this: (1) determining the prestige and status of languages; (2) standardising and perpetuating language ‘correctness’, and (3) suppressing language diversity. My interest in this article lies in how the GPS tests cut across these, but with a particular focus on (2), in the ways that they might intimidate teachers into pedagogies that they do not necessarily value or believe in, uncritically advocate standardised English at the expense of nonstandardised forms, and frame language as an ideological system of ‘correctness’ imbued with notions of superiority, hierarchy and legitimacy (Bourdieu 1991).

There are various features of tests which create the impression of power. These include material aspects of the test papers, including the use of numerical language, instructions, logos and symbols, but also the procedural, ceremonial devices such as the layout and atmosphere of the test room, and the ‘secretive machinery’ of the testing organisation (Shohamy 2013: 228), which in the case of this article, refers to the UK Department for Education (DfE) and the Standards and Testing Agency (STA). One attraction of language tests to governments lies in them being perceived by the public as authoritative, being effective for controlling linguistic knowledge, crafting impressions of objectivity, and allowing for cost-effective policy making. Language tests can appear to show short-term gains because they provide data which can be used by governments as a proxy for improved literacy rates, a policy move which has a long history in England (e.g. Moss 2009; Wyse and Torrance 2009). However, as Lingard (2012) demonstrates in relation to the Australian National Assessment Program, high-stakes tests can have damaging effects in the long-term, in terms of narrowing pedagogies and curricula to become overtly test-focused and degrading teachers’ work (see also Menken 2008 for a critical discussion of the *No Child Left Behind* education policy and tests in the USA).

‘Testers’ and ‘test takers’ are important terms within critical language testing, referring to a range of people and organisations across policy layers (see Shohamy 2013: 227–228). At macro-level, *testers* are typically employees within government departments who make decisions to implement tests and evaluate their impact. The DfE and the STA are the government bodies in control of the GPS tests, with two education ministers in particular, Michael Gove and Nick Gibb, at the spearhead of the testing agenda. Gove and Gibb’s roles as policy actors is explored further below. At meso-level, testers include school management, who are required to ensure that teachers teach the content that the tests demand. At micro-level, classroom teachers are held responsible for preparing students for tests. *Test takers* refer not only to students, but also to others who are in some way involved in the testing regime or held accountable in some way: typically, teachers, parents and school management. This article is primarily concerned with the effects of the GPS tests on teachers, as policy actors who can be manipulated into pedagogies which reproduce the language ideologies embedded within the tests.

## Methodology and data

This article combines Shohamy’s model of critical language testing with critical discursive approaches to language policy as a way of understanding the power and social life of the GPS tests. There are four steps in the model, which track a test through from conception to consequence and policy to practice (see Shohamy 2001: 106–107). Step 1, *origins,* requires analysts to examine the relevant socio-political contexts which led to the introduction of a test, tracing the educational conditions in which a test is conceived of and created, and in doing so, considers tests as a political and ideological tool. Step 2, *manipulations*, involves examining test questions, in how they might be used by authoritative bodies as tools to introduce agendas and enforce policies, and to consider what kind of language ideologies might be embedded within them. Step 3, *effects*, attends to the effects and impacts of tests in terms of the kind of pedagogies, discourses and resources teachers might implement in response to them. This involves looking closely at policy enactment and mechanisms such as pedagogical materials and curricula. Step 4, *consequences*, examines the broader societal and educational consequences of tests, asking questions around how tests shape policy actors’ perceptions of language and education. Taken together, the steps in the model offer one way of revealing the trajectory of language ideologies found throughout policy layers. Tracing this trajectory requires the generating and triangulating of a range of data types and sources, which are outlined in Table 1.

**Table 1:** Sources of data across policy layers

Data; policy layer	Rationale for inclusion
Political discourse by two key government ministers in the GPS agenda, Michael Gove and Nick Gibb. Further information about these policy actors is provided below. 30 speeches were collected, delivered at teacher conferences, education summits and schools, from across 2009–2019. These speeches were largely used to either frame the introduction of national education reforms or provide ongoing claims about their efficacy. All speeches in the dataset were selected on the basis that they included discourse related to grammar, English teaching or the GPS tests (e.g. Gibb 2015; Gove 2010)	To trace the origins and emergence of the tests and to consider how they fit within a longer historical narrative of language in schools. To explore the trajectory of the tests across a decade of c/Conservative involvement in education reform, and to interrogate the tests as part of this political agenda
White papers and reports published by the government which refer to the tests and curriculum reform (e.g. DfE 2011a; House of Commons 2017a)	To examine how the tests were conceptualised and legitimised within curriculum policy
Guidance documentation for test developers (STA 2015)	To examine the guidelines and constraints under which test developers operate
The GPS test papers and mark schemes, from their pilot in 2013 through to the 2019 papers (e.g. DfE 2019a). The 2020 tests did not go ahead due to Covid-19	To examine the language of test questions and their potential to steer teachers towards certain pedagogies and the reproduction of language ideologies
National curriculum framework for primary schools in England (e.g. DfE 2013a)	To examine how the tests are part of a broader curriculum policy and how they are used as a vehicle for its implementation
An online survey distributed to primary school teachers in England, in which they were asked about their perceptions of the tests. This received 78 responses. The survey included 11 questions, organised into three sections: (1) demographics and teaching experience; (2) perceptions of the tests and their impact, and (3) school-designed language policies. It was open for a period of three months, in Autumn 2019, and advertised over social media channels and on teacher web forums. Only teachers who had taught the content of the tests were invited to respond, although I had no control over this given the openly accessible nature of the survey. It may of course be true that only those who held particularly strong views about the tests were motivated to respond, and this should be considered as a limitation of the data. The details of survey respondents are available in Appendix A	To determine a broad picture of teachers’ views on the tests and their impact, positioning teachers as crucial negotiators of language policies (e.g. Menken and García 2010)
Semi-structured interviews with 19 practicing primary school teachers in which they were asked about their perceptions and lived experiences of the tests. Participants were identified through the survey, and by responding to a call for participation which was placed on social media channels. Interviews lasted 20–45 min, were audio recorded and professionally transcribed. The interview guide was organised around the same three sections used in the survey. Again, a potential limitation of this data is that only those who held particularly strong feelings were motivated to participate, yet this is typical of sociolinguistic interviews of this nature. The details of interview respondents are available in Appendix B	To examine teachers’ views about the impact of the tests in further detail, and to understand how the tests—as one arm of a policy mechanism—can manipulate pedagogies and potentially work as a vehicle for macro-level language ideologies

### Data preparation

All policy documents, political speeches, interview transcriptions and the survey results were indexed using NVivo software. This began to make sense of the dataset, in organising the data into themes under different coded headings and making visible trends and contact points across different sources of data and policy layers. In taking a pragmatic process to coding, I began with some broad themes or ‘parent codes’ relating to language and educational ideologies, policies and tests, out of which emerged sub-themes or ‘child codes’. The coding process took a number of months, involving retracing and checking codes through the dataset. Out of this, a final framework was built, and the most prominent codes were used to steer the organisation of the analysis sections which follow.

## A critical discursive analysis of the tests

This section examines the power of the tests, tracing discourses about them through policy layers and toggling between different sources of data. I begin with an analysis of the political discourse which framed their implementation, before analysing test questions themselves, and then turning my attention to teacher discourse about the tests. The headings of the subsections that follow mirror the steps outlined in Shohamy’s model: origins, manipulations, effects and consequences.

### Origins and political framings

This history of the GPS tests begins with data taken from a speech delivered by Michael Gove on the 30 June 2009, who at the time, was the Shadow Secretary of State for Children, Schools and Families. In UK politics, Gove is known for his nationalistic, neoconservative ideologies and voting history, being at the spearhead of the 2016 EU membership referendum and the chief architect of 2010 education reforms, despite having no experience of working in schools. I begin here because it is illustrative of Gove’s rhetoric during this period (see Jones 2014: 98–99), in which he called up the kind of language ideologies which framed the introduction of the original national curriculum in the 1980s, criticised twelve years of ‘progressive’ Labour education policy and set out his own agenda for a ‘traditional’ curriculum, part of which included children having access to ‘intellectual capital’ such as standardised English to help ‘bind society together’ (Gove 2009). As detailed in Wyse and Torgerson’s (2017: 1024–1027) history of post-2010 education reforms and the emphasis on grammar within these, Gove’s policy moves were based on ideology and a dismissal of academic ‘experts’ rather than research evidence. Gove’s visions of educational policy were primarily derived from E.D Hirsch, who in his *Dictionary of Cultural Literacy: What Every American Needs to Know*, lists 5000 ‘facts’ that he sees as an ‘essential body of knowledge’ in order to be ‘literate’ (e.g. Gove 2014). This knowledge is framed as intellectual capital for nation building, including standard language ideologies such as how ‘fixing the vocabulary of a national culture is analogous to fixing a standard grammar, spelling and pronunciation’ (Hirsch 1987: 84; see Blackledge 2009 for one critique of this ideology). There are numerous references to Hirsch in the dataset of Gove and Gibb’s speeches, and he became a prominent policy actor in the discourse of 2014 curriculum reform, with critics arguing that his ideas about language, society and culture are rooted in prescriptivism, xenophobia and cultural elitism (e.g. Yandell 2017). In the same speech, Gove hinted at the GPS tests by declaring that he would ‘reform our SATs to sharpen accountability and drive up standards’, including the explicit appreciation of standardised English and canonical, British literature. The origin of the tests then, was as a buttress for the standards discourse which has permeated English educational policy across successive governments since the introduction of the national curriculum in the 1980s.

Gove became the Secretary of State for Education in 2010, appointing Nick Gibb as the Minister of State for School Standards, a role which bears ultimate responsibility for SATs and their administration. Similar to Gove, Gibb has no experience working in schools, aligns himself with Hirsch’s right-wing educational ideologies (e.g. Gibb 2015) and is an advocate supporter of high stakes testing as a sole indicator of educational success (Gibb 2017). Throughout the dataset and revealed through the coding process, Gove and Gibb’s speeches work to (re)construct crude oppositions between so-called ‘traditional’ and ‘progressive’ education, criticising ‘progressive’ notions such as ‘exploratory learning’ whilst championing ‘traditional’ notions such as ‘discipline’, ‘grammar drills’, ‘teacher-led instruction’ and ‘formal testing’. Such discourse typifies the dense ideological web in which debates about grammar take place, continuing the politicisation of language in English schools in which ‘good grammar’ is used as a proxy for ‘good behaviour’ (see Cameron 2012: 78–115). Gibb made claims of ‘a steady but remorseless decline in standards’ (2017), ‘falling societal standards’ and ‘out of control classroom behaviour’ (2010), whipping up accusations of language and disciplinary decline which required drastic and remedial policy intervention. At the 2010 Conservative party conference, Gove announced his plans to reintroduce ‘proper’ grammar teaching and testing to the curriculum, using a supposed ignorance of grammar in schools as an opportunity to point criticism at the previous government’s language education policies:

Thousands of children—including some of our very brightest—leave school unable to compose a proper sentence, ignorant of basic grammar, incapable of writing a clear and accurate letter. And it's not surprising when the last Government explicitly removed the requirement to award a set number of marks for correct spelling, punctuation and grammar in examinations. The basic building blocks of English were demolished by those who should have been giving our children a solid foundation in learning. Well—let me be clear. Under this Government we will insist that our exams, once more, take proper account of the need to spell, punctuate and write a grammatical sentence. (Gove 2010).

These plans first took shape in a White Paper which correlated ‘correct grammar’ with employment prospects (DfE 2010: 49), and then with the publication of the Bew Report^2^ (DfE 2011a, b). The focus of the Bew Report was on testing, assessment and teacher accountability, and whilst an interim report made a fleeting reference to the possibility of grammar tests (DfE 2011a: 30), the final report which appeared three months later (DfE 2011b) included the recommendation that grammar ought to be tested on the grounds that there are ‘right’ or ‘wrong’ answers:

We recognise there are some elements of writing (in particular spelling, punctuation, grammar and vocabulary) *where there are clear ‘right or ‘wrong’ answers*, which lend themselves to externally-marked testing. **We recommend that a test of these essential writing skills is developed**. (DfE 2011b: 14, original emphasis in bold; my emphasis in italics).

In 2011, a working group of linguists and educationalists was constructed by government to review such recommendations, which came to raise concerns around reductive ‘right/wrong’ grammar (Myhill, personal communication; see also STA 2013: 13–14). Some members of the working group would later reveal the ‘chaos’ involved in the development of the tests and have since called for them to be ‘scrapped’ (see Mansell 2017). Crowley’s (2003) history of grammar in England’s schools reveals similar policy moves during the introduction of the original national curriculum in 1988, with education ministers foreshadowing Gove and Gibb’s yearning for ‘old-fashioned grammar’ and ‘proper language’. In this way, the grammar tests can be seen as an explicit attempt by Gove and Gibb to continue and complete the work of their predecessors in the 1970s–80s, using tests as one mechanism to do so.

The tests were formally introduced to schools in 2013 as part of a wider curriculum reform, accompanied by a technical report (STA 2013), a detailed document which is characterised by the language of testing and statistics, in what Ozga (2009) conceptualises as governance through a ‘regime of numbers’. Gibb (2016) championed the new curriculum on the grounds that the tests and the accompanying grammar glossary—an eighteen-page document of 60 clause-level grammatical terms (DfE 2014)—would aid students’ learning of English, despite a wealth of evidence suggesting that decontextualised grammar does nothing for students’ literacy abilities (e.g. Myhill and Watson 2014). Gibb’s support for the tests was framed through his idea that testing is ‘vital’ and should be a ‘normalised part of school life’ (Gibb 2016). As such, they were introduced as one mechanism of pedagogical control, presented by government as an authoritative measurement of student success and teacher performance. Indeed, the tests do not just test children’s knowledge of grammatical terminology—they test teacher’s abilities to teach it, framed as the most ‘reliable’ assessment of teachers by government (DfE 2011b), but here conceptualised as a policy technology of surveillance (see Page 2017) which monitors, categorises, praises and punishes teacher performance.

In 2017, an inquiry at the House of Commons Education Committee (2017a), raised concerns about the tests to government. Using evidence submitted by 388 teachers, academics and parents, the committee argued that the tests were damaging the well-being of test takers and questioned the assessment of decontextualised grammatical knowledge. Despite the evidence, the government rejected these concerns (House of Commons 2017b), citing an Office for Standards in Education report which recommended an *increase* in grammar teaching (Ofsted 2012), partly due to the ‘lack of emphasis’ on standardised English in schools (ibid. 54). Since then, teachers, parents and community activist groups such as *More Than A Score* have commissioned a number of research reports (e.g. Bradbury 2019) focusing on the damaging impact of SATs, as well as leading nationwide school strikes and test boycotts. Other activist groups working across the UK, such as *Let Our Kids Be Kids*, *Save Our Schools* and *Reclaiming Schools* engage in similar activities. Although strikes and boycotts send a clear message to macro-level policy makers, education ministers such as Nick Gibb have dismissed such activism as a ‘undermining’ curriculum reform and teachers’ work (Weale 2019). Gibb was equally dismissive of a 2019 poll conducted by the National Education Union which saw 97% of primary school teachers voting to indicate their preference for SATs to be abolished.

This section has argued that the GPS tests were introduced by the Conservative government as a buttress to support their own educational and linguistic ideologies, underpinned by notions of discipline and standards which continues a longer historical narrative in which deficit language ideologies of ‘correctness’ come to shape policy. Discourse from government policy actors and materials reveals how the tests are championed by politicians under the guise that they improve literacy abilities, despite a lack of research evidence to suggest that decontextualised language assessments do this.

### Manipulations: the tests

This section examines the GPS tests themselves. The requirements for test conditions are laid out in a 34-page document (DfE 2019b): students to be in silence, sat on isolated tables, not to ask questions, to perform under a time constraint, and for teachers to be monitors. DfE and STA branding and the language of numbers appear throughout the test papers, carrying symbolic power in the sense that this makes the tests appear trustworthy, whilst reducing linguistic knowledge to a numerical abstraction. Students are required to answer five different question types, with an overview of each shown in Table 2.

**Table 2:** Question types in the GPS tests

Question type	Rubric subtype	Examples
Identify	Test pupils’ knowledge of linguistic terminology by identifying the correct response	Identify which sentence is written in Standard English Circle all the [nouns] in the sentence below
Complete/correct/rewrite	Insert or generate a specified type of response within a given structure, either to complete it or correct an error in it	Rewrite the sentence below, changing it to [past tense] Circle the incorrect [verb form] in the sentences below
Match	Require the pairing of two different elements	Draw lines to match each sentence to [its type]
Write	Require pupils to generate their own examples of specified language, or to label language with a technical term	Write a statement in response to the question below
Explain	Require pupils to demonstrate their understanding of particular linguistic terms	The sentence below has [an apostrophe] missing. Explain why it needs [the apostrophe]

These question types reproduce a prescriptive language ideology, where the focus is on identifying grammatical terms in sentences written in standardised English and correcting nonstandardised ‘errors’. ‘Language’ is here constructed as a written, clause-level system, governed by standardised ‘rules’ which bear little resemblance to everyday discourse. I analysed all of the available test questions (*n* = 462), assigning each token to one of the rows in the taxonomy in Table 2. The results of this are shown in Table 3.

**Table 3:** Frequency of question types across the 2014–2019 papers

Question type	Frequency
Identify	269
Complete/correct/rewrite	134
Write	35
Match	15
Explain	9
Total	462

**Figure 1:**
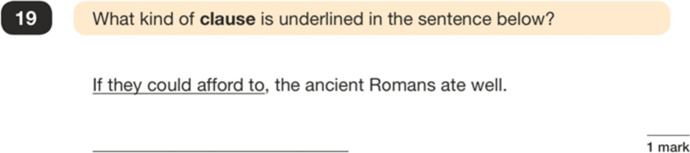
An ‘identify’ question type (example 1)

The dominance of ‘identify’ and ‘complete/correct/rewrite’ question types (87% of tokens across 12 test papers) indicates that test questions are designed to be marked quickly and economically efficiently—framing language as a list of technical terms, and something that can be assessed in numerical, unambiguous ways. In contrast, the limited number of ‘explain’ questions (2% of tokens) provides very few opportunities for students to engage with language in evaluative or descriptive terms. An example of an ‘identify’ question type is shown in Figure 1.

**Figure 2:**
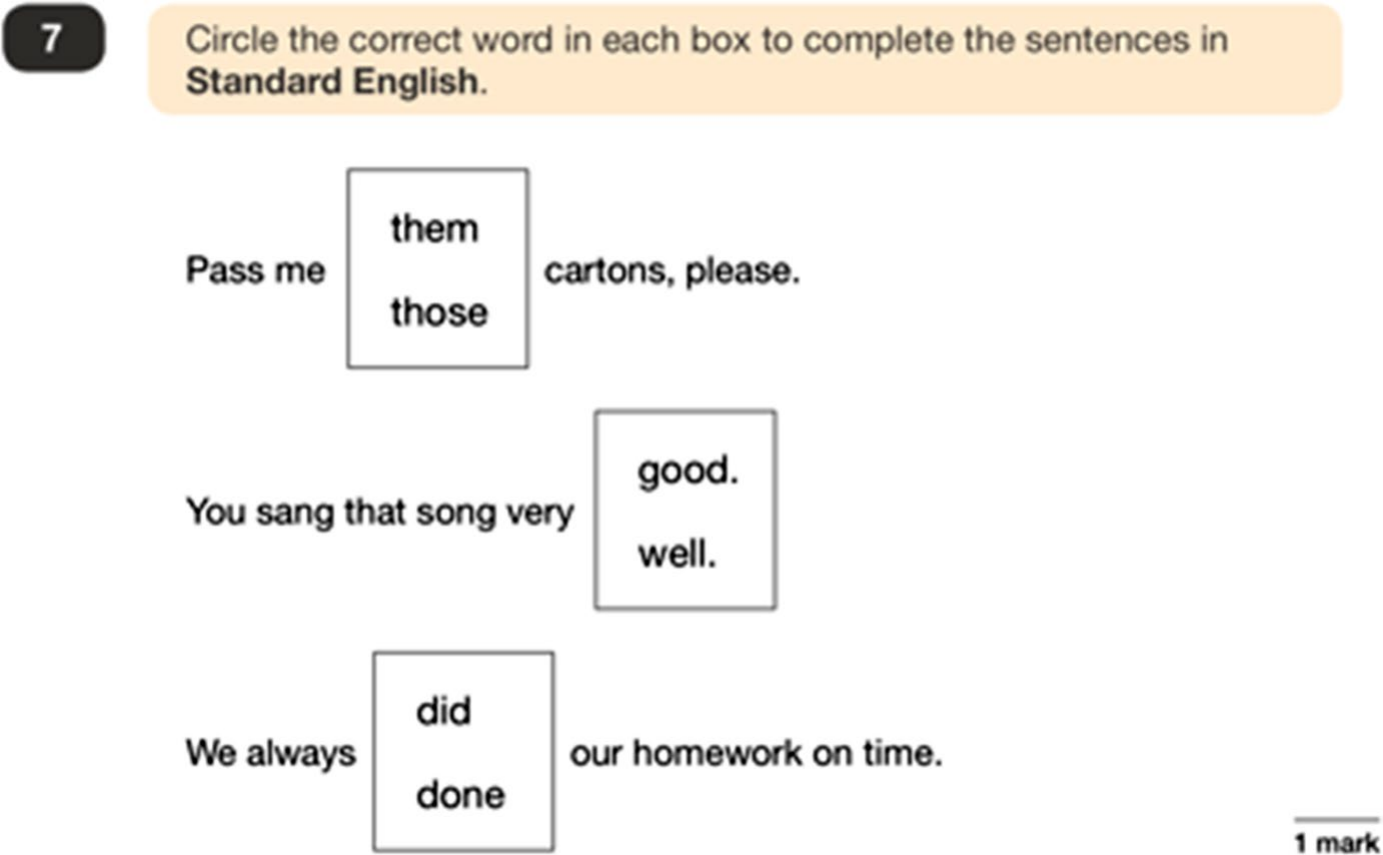
A ‘complete/correct/rewrite’ question type (example 1)

**Figure 3:**
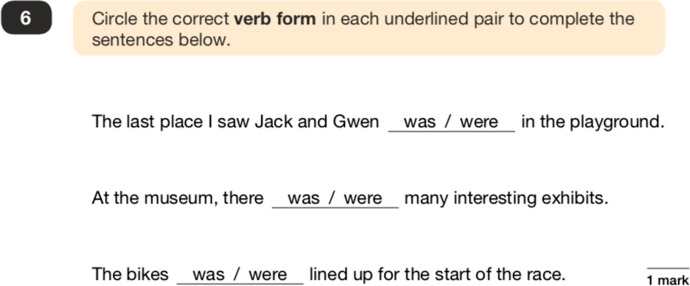
A ‘complete/correct/rewrite’ question type (example 2)

Other ‘identify’ questions follow a similar format, with students being asked to demonstrate their knowledge of 40 metalinguistic terms as defined in the curriculum, drawing from a narrow and ‘traditional’ framework (Bell 2015). Such ‘naming of the parts’ questions resemble school-based grammatical work from the 1950s (Crystal 2017) with scant evidence to suggest that this kind of activity bears any positive effect on writing or literacy abilities (see Myhill and Watson 2014). The examples of language in the test questions is awkward and robotic, designed purely for testing purposes, and a far removal from how real language is used in everyday discourse. Given this, the questions leave no room for test takers to consider the nature of language as a social system of communication. Two examples of a ‘complete/correct/rewrite’ question type are shown in Figures 2 and 3.

Questions such as these foreground a particular language ideology around cultural linguistic bias (Milroy and Milroy 1991: 166–175), namely the idea that standardised English is the ‘correct’ form of the language, that nonstandardised forms are not tolerated, and that language can be reduced to numbers which are easily assessed in an education ‘assembly line’ (Au 2011: 36–38). Figures 2 and 3 use nonstandardised grammatical constructions as examples of ‘incorrect’ language, which potentially work to socialise children into labelling what might be legitimate features of their own dialects as ‘wrong’. For example, in Figure 2, the use of ‘them’ as a determiner (as in ‘them cartons’) rather than its prototypical use as a pronoun (as in ‘can I see them?’), is a legitimate grammatical construction found within many UK varieties. Figure 3 is a typical example of how auxiliary verb ‘was/were’ variation is used in the tests as a way of promoting the illusion of a ‘right/wrong’ dichotomy in language, underpinned by value judgements which potentially work to stigmatise speakers of the many UK varieties which use this nonstandardised form.

Recent work in educational sociolinguistics (e.g. Cushing 2020a, b; Snell 2018) has shown how racialised and classed structural language stigma is increasingly normalised in England’s schools, partly as a result of post-2010 educational reforms which emphasise the requirement for teachers and students to use standardised English in schools. GPS test questions are here positioned as policy mechanisms which contribute to this stigma, reproducing an ‘imposition of uniformity and the imagined dichotomy between ‘standard’ and ‘non-standard’’ (Milroy 2001: 346). The guidance for test developers (STA 2015) specifies the nonstandardised constructions which are deemed to be ‘incorrect’, revealing the value-laden benchmark which children are assessed against and further entrenching the standard language ideology. As well as intra-language variation, test guidance only permits British English conventions to be permissible answers in test questions, dismissing inter-language variation and ‘non-British’ varieties of English (STA 2015: 15). Such stipulations put speakers of languages other than English at a clear disadvantage, promoting not just ‘English only’ policies, but ‘*British* English only’ policies, an ideology which is wrapped up in the Anglocentric nature of post-2010 curriculum reforms as discussed earlier. Whilst British English is framed as carrying linguistic capital and as a gatekeeper to earning ‘credits’ in tests; non-British varieties and their speakers are devalued, framed as ‘wrong’, marginalised and discredited (see Blackledge 2009). It is perhaps ironic then, that the same STA policy document claims to present a test which ‘provide(s) opportunities for all pupils to achieve, irrespective of […] social, linguistic or cultural backgrounds’ and ‘be free from stereotyping and discrimination in any form’ (STA 2015: 31).

The message about language, delivered via the tests, is that written, standardised, British English is the exclusively legitimate variety of the language, with any deviations from this being devalued, deviant and defective. The tests serve as a textual vehicle through which standard language ideologies are communicated to test takers: as ‘tools to privilege certain forms […] of language knowledge’ (Shohamy 2007: 120), and as powerful artefacts which have the potential to control and manipulate pedagogies. The ‘power’ of the tests in this sense is explored in the following section.

### Effects, consequences and pedagogical coercion

This section explores the power of the GPS tests in their ability to control pedagogies and manipulate teachers into reproducing the language ideologies which underpin them. These were the most prevalent themes to arise out of the coding process. Data here are drawn from the surveys and interviews as outlined in Table 1.

Teachers’ perceptions were largely negative, with participants talking about their dislike of the tests, the ways in which language was conceptualised as a narrow, artificial body of study, the damaging impact on writing, the large amounts of curriculum time spent preparing for the tests, feelings of reduced autonomy and threats to professional identity. Although the data present a negative evaluation of the tests, caution must be taken here in that teachers volunteered to take part in interviews, and so represent a pool of particularly motivated participants to share their thoughts. Nevertheless, these thoughts are valid and motivated by a desire to express their frustration at what they felt to be a testing system which negatively impinged upon their agency. The data reveals the power of the tests in serving to regulate teachers’ behaviours and choices, in what I define as *pedagogical coercion*: a process whereby teachers’ practice comes to be distorted by macro-level pressures and language policy mechanisms which are felt to be more powerful, socialising and intimidating teachers into actions that they do not necessarily believe in or value. Some initial examples from survey respondents illustrate this, where I have also included the year that the teacher took responsibility for (Year 6 denotes the final year of primary school, during which the tests are taken):

My teaching of grammar is now all purely focused on the test and making sure that the students know the terminology and how to identify it. It’s not teaching as I want to or know it—it’s teaching to the test, pure and simple. They make me do things I don’t want to do. (Survey response #16, Year 6 teacher).

I feel gripped by the test and what it makes me do. It has reduced my teaching to a box-ticking exercise. (Survey response #35, Year 5 teacher).

The tests have made my job torturous. It’s like making people who are learning to drive strip down a car engine with their bare hands. (Survey response #07, Headteacher).

These responses demonstrate the power of the tests as de facto language policy, or, in terms of Shohamy’s framework, their *effects* and *consequences*. There are physical and violent metaphors here of control, with teachers talking about the agentive power of the tests in how they manipulate pedagogies (e.g. ‘I feel gripped by the test’; ‘they make me do things I don’t want to do’) and have led to bleak perceptions of teaching itself (‘they have made my job torturous’). Many of the comments made intertextual connections to macro-level policies and political figures who bear responsibility for the tests, revealing textual trajectories of government language ideologies into classrooms (see Cushing 2020b; Johnson 2015). For instance, in comments made by teachers both with over 10 years of experience:

I think you’ve got a whole community of teachers now who really fundamentally disagree with how things are tested, who totally disagree with everything they’re being made to do by government but feel utterly disempowered to do anything about it. (Amy, Headteacher).

The government seem to do all they can to destroy education and suck the joy out of primary schools. They seem intent on forcing onto us stuff about language which seems so false, and they just have a very stale view of grammar teaching and testing. I cannot bear to even think about Nick Gibb and Gove. They’ve had such a damaging effect on my classroom. (Iris, Year 5 teacher).

The data suggests that despite many teachers not valuing or believing in the pedagogies they were enacting, there were high degrees of constraint, largely because of the felt power of the tests, teacher accountability and the view of grammar contained within them. Responses from the survey reinforced this, with comments suggesting that the tests are, for example, ‘very powerful’, ‘destructive’, ‘[having] the power to dictate how grammar is taught’, and ‘a tool to beat teachers with’. For the teachers who reported these feelings, they are relegated to a position which distributes macro-level policies despite their voiced concerns and resistance (Fitzsimmons-Doolan 2019), coerced into pedagogies that exist to provide assessment data rather than to create meaningful experiences for the students they teach. Although the ‘macro–micro’ distinction is an over-simplification, it is nonetheless a convenient metaphor for understanding how agents have different amounts of autonomy and power at different policy levels. Carl, an assistant headteacher with 20 years of experience, and Alex, a teacher with 7 years of experience, provide two further illustrations of this:

[The curriculum] has been narrowed in two ways; one in literally time so any schools, there are lots of schools doing it, any time that’s given over, half an hour every day to grammar just to prepare for those tests. Totally separate to the English lessons that would normally take place, where you know things like reading and writing would be happening, storytelling, that kind of thing. It is narrowing the curriculum because there’s still too many children who don’t read and don’t write and the tests just distort their view of English and language. They create a false kind of subject which I don’t recognise as English. (Carl, Year 6 teacher).

The tests and the curriculum […] are soul-destroying. (Alex, Year 4 teacher).

Carl’s spatial metaphors to describe the curriculum (e.g. ‘narrowing’, ‘separate’) indicate that the tests have had a physical impact on teaching in his school, with an increased amount of time dedicated to test preparation and decontextualised grammar, at the expense of other things which he deems to be more valuable (e.g. ‘reading’, ‘writing’, ‘storytelling’). Vision metaphors (e.g. ‘distort their view’) suggest the warping effect that the tests have on his curriculum and his pedagogies, whilst Alex’s ‘soul-destroying’ comment frames the tests as a powerful force which physically threatens his professional identity. Evidence that teachers are coerced into ‘teaching to the test’ was prevalent throughout the data, reflecting Bradbury’s (2019) work which surveyed and interviewed headteachers about their feelings towards primary school SATs more broadly. Data in Safford (2016) suggested similar findings, with teachers reporting that the tests have prompted wholesale changes in pedagogies which are governed solely by the nature of the test questions. For many teachers in the current study, time spent preparing for the tests felt ‘punitive’ (Keisha) and ‘futile’ (Alice), often because of feelings that the content was being taught purely for testing, accountability and surveillance purposes. This ramping up of test preparation work was found to be especially true in the final two years of primary school, with a number of Year 6 teachers talking about the ‘freedom’ they had once the tests had been taken in May.

The previous section explored the decontextualised nature of the GPS tests, and the foci on ‘error correction’ and ‘feature spotting’. Data from participants was clear in that this often resulted in pedagogies which were governed by this way of conceptualising language, where ‘artificial’ language work tended to take place:

And the format of the test is not about understanding, it is a feature spotting activity. I think the grammar teaching I end up doing is really quite artificial because it’s all about underlining features and using overly technical terms just for the sake of doing so. It turns into a box ticking exercise. I wonder is it doing anything useful? I don’t think so. It’s not teaching anything about language apart from the names of little tiny bits. (June, Year 4 teacher).

There has been so much more decontextualised teaching because of the tests. I’m forced into doing it to prepare students for the test because that’s the grammar that they need to be familiar with to get through the test. (Carl, Year 6 teacher).

What the tests have led to, which I don’t necessarily agree with, is a lot of standalone grammar teaching where children complete worksheet exercises or tick box things or fill in the gaps to learn this grammar technique. […] What I then sometimes don't see is that knowledge crossing over into the writing which is actually where the grammar is the most important. (Billy, Year 6 teacher).

Here, teachers talk about the emphasis on the ‘identify’ question styles as discussed in the previous section—and the fact that the test design has coerced them into pedagogies which focus on the identification of clause-level grammatical metalanguage. Teachers were generally critical about the emphasis on grammatical terminology in the tests, questioning the value of this knowledge in regard to developing literacy abilities. Some participants talked about how they were resisting the prescriptive ideologies of the tests and employing contextualised grammar pedagogies which were focused on authenticity, description and meaning, negotiating language policy in ways which is agentive and creative (Menken and García 2010). However, this was often presented as being ‘subversive’ and talked about in terms of desire rather than reality:

I do want to teach grammar in context and get away from the rote learning of the test. But my school insists on discrete grammar lessons where we have to teach them what is ‘right’ and ‘wrong’ and so I end up doing that (Survey response #17, Year 3 teacher).

[The tests] remove the focus on creativity from writing and reduce it to a ‘paint-by-numbers’ approach to construction and assessment (Survey response #02, Year 4 teacher).

For many teachers, writing under the GPS agenda has become an artificial exercise where the focus is on inserting grammatical features in order to score marks. This was a prevalent theme of the surveys and interviews, with teachers talking about how the emphasis on decontextualised grammatical terminology coerced them into pedagogies to the detriment of children’s writing quality and their enjoyment of it. These comments are particularly interesting in that they directly challenge Nick Gibb’s insistence that the tests are improving writing (Gibb 2018), which was one of the government’s original stated motivations for introducing the tests (see DfE 2011b: 14). Gibb claimed that the tests have improved writing on the simple basis that the national average of test results has steadily increased, pointing to England’s rise in international league tables as an indicator of ‘progress’. However, critics (e.g. Barrs 2019; Hardman and Bell 2019) have shown that in reality, these ‘improved’ writing scores are simply a blunt measurement of how well children are able to insert arbitrary grammatical features into their writing in formulaic approaches, rather than a nuanced, genuine measure of writing quality and competence. ‘Standards’ here then, are based on crude notions of language awareness, measured by test scores which is typical of neoliberal education systems such as those espoused by the Conservative government (see also Pratt 2016 for a UK-based primary school case study of how assessments manipulate pedagogies in accordance with market-driven demands and pressure).

In earlier sections, I showed how a cluster of policy mechanisms such as political discourse, curriculum documents and the GPS tests textually reproduce the standard language ideology. Interview and survey data revealed how teachers can be socialised into acting as vehicles for these ideologies, for instance:

I hate the focus on standard English in the test questions, the stuff about formal language and correcting errors you know, just hate it. My pupils don’t speak like that and why should they? But to pass those tests I have to tell them how to speak and so you know. It can feel so fake. (Xena, Year 5 teacher).

We’ve got no choice because of the content of the tests, they just have to be able to speak in a certain way and use language in a certain way, and that’s definitely dictated by the tests and the glossary and things. (Lucy, Year 5 teacher).

The way that the tests describe language is so fake. I know that language doesn’t work like that but I’m kind of steered into saying that it does in my classroom because of the language of the tests. (Carl, Year 6 teacher).

There is a clear discourse here about the way that teachers are coerced by the tests into constructing standardised English as a punitive system of control and surveillance. Verbs such as ‘I *hate* the focus on standard English’, ‘I *have* to tell them’ and ‘I’m kind of *steered’* suggest that even though they may resist it, teachers can be coerced into acting as a vehicle for standard language ideologies underpinning the tests and the curriculum. Xena, a teacher working in an economically deprived area of Northern England, knows that the version of language she is compelled to teach is ‘fake’ and not representative of how her students speak but positions herself as powerless in reference to the felt pressure that the tests create. This resonates with Crowley’s (2003) critical history of language in schools, where he argues that teachers functioning as a mouthpiece for a government’s language ideologies has long been the case in UK education. Data here would support this in reference to contemporary language policy, especially in the ways that teachers can be positioned by policy mechanisms as regulators and managers of language in schools (Cushing 2020a; Spolsky 2009).

## Conclusion

This article has presented an analysis of the GPS tests, through a broad lens of discursive approaches to language policy and using Shohamy’s (2001) framework of critical language testing, which sees tests as non-neutral products of political and ideological agendas. Although SATs and testing regimes have long been politicised in England (e.g. Marshall 2017), this article has been the first to examine the political agenda which underpins the GPS tests. Drawing on a range of data types and sources which reach across policy levels, I have shown how the tests work as de facto language policy, designed to assess an oppositional, ‘right-wrong’, binary version of language which bears little resemblance to the linguistic repertoires of its test takers. The research has illustrated the ‘impact of coercive policies on language learning and language use’ (Tollefson 2015: 140), showing the distortive power of an ideologically-driven mandated testing regime which can deprofessionalise teachers and constrain their pedagogical autonomy. This process of pedagogical coercion occurs within an education system where teacher agency undoubtedly exists, but can be curtailed by various mechanisms, including the GPS tests, and additional pressures such as management, inspection bodies, curriculum organisation and teacher surveillance. Technologies of teacher surveillance saturate England’s education system (e.g. Page 2017), and here the GPS tests can be seen as one mechanism which works in this way. Indeed, the GPS tests do not just test students, but test teachers’ abilities to ‘deliver’ top-down content which is driven by standard language ideologies and racialised, archaic notions of ‘proper grammar’. Data revealed that these pressures to comply with policy were especially pertinent for teachers in Years 5 and 6 of primary school, as curriculum time started to be narrowed towards test preparation. The emphasis on standardised English at macro-level policy and the lack of critical reference to its social power serves to frame nonstandardised forms as deficit varieties, with the tests functioning as one ‘practical implication of prescriptivism’ (Milroy and Milroy 1991) which can further entrench hierarchical, standard language ideologies.

Tracing the steps within Shohamy’s model of critical language testing has shown how the GPS tests can work as de facto language policy in English schools. In terms of *origins*, the tests were introduced as one arm of the Conservative government’s curriculum reforms, used as a buttress to support a (re)shift towards policies underpinned by a nostalgia for ‘tradition’ and ‘standards’. They are a particularly iconic symbol of Michael Gove and Nick Gibb’s policy agendas, used as one attempt to assert their authority within their roles in the formation of a new government. Analysis of the test questions and assessment frameworks revealed the embedded *manipulations*, which frame language as a crude system of ‘rights’ or ‘wrongs’ and place an uncritical emphasis on standardised English which serves to delegitimise nonstandardised variation. Test questions assess decontextualised language knowledge which focuses on language ‘identification’ and ‘correction’, failing to recognise the social and communicative dimensions of language. Data from teachers showed how these ideologies can get reproduced in practices, and so in terms of *effects* and *consequences*, the tests are extremely effective in governing classroom activity and shaping the type of knowledge about language that children engage in. Furthermore, data explored in this article has shown how the GPS tests can coerce teachers into pedagogies that they do not believe in or value, functioning as a threat towards their professional identities and epistemologies, and often reported using violent metaphors of physical manipulation and control.

There are, of course, limitations to this paper, which future research might seek to address. A further way of tracing language ideologies from policy to practice would involve an ethnographic exploration of how a school or schools were enacting the GPS tests, rather than attempting to elicit a more general picture, which this article has offered. Data from this article revealed strong, negative feelings towards the tests, but caution must be taken here in over-generalising about the entire teaching population. Granting too much power to tests and top-down policies is an ongoing criticism of language policy work (e.g. Menken and García 2010), and although the research presented in this article has suggested that the GPS tests do indeed carry regulatory power despite teachers’ concerns, work which looks at the creative ways in which teachers resist and negotiate these tests in their own micro-level policy spaces would be of value. Work of this nature, which highlights the nuanced and agentive aspects of policy making within schools, would be a welcome addition to UK-based critical language policy research in further exploring the intersections of testing, accountability and language ideologies.

## Data Availability

The data set associated with this paper can be found at the following location: 10.17633/rd.brunel.c.4720436 Grammar tests, de facto policy and pedagogical coercion in England’s primary schools.

## Appendix

Demographic information for research participants.

## Appendix A: Survey participant information

**Table:**

Experience in years	*n*
0–5	13
6–10	29
11–15	21
16–20	11
20 plus	4

Main position	*n*
Teaching assistant	5
Teacher	49
Teacher w/ other responsibility	14
Headteacher	9

Current Key Stage	*n*
KS1	21
KS2	57

## Appendix B: Interview participant information

**Table:**

Experience in years	*n*
0–5	1
6–10	9
11–15	5
16–20	2
20 plus	2

Main position	*n*
Teaching assistant	0
Teacher	10
Teacher w/ other responsibility	4
Assistant headteacher	3
Headteacher	2
Other	0

Current Key Stage	*n*
KS1	4
KS2	15

## Footnotes

1 Office for Standards in Education, Children’s Services and Skills. In England, Ofsted carry out school inspections and grade them in one of four ways: ‘outstanding’, ‘good’, ‘requires improvement’ or ‘inadequate’.

2 Paul Bew is a professor of politics, with close links to the right-wing think tank Policy Exchange, for which Nick Gibb has written for about his admiration of E.D Hirsch (Gibb 2015).
